# The probiotic strain *Escherichia coli* Nissle 1917 prevents papain-induced respiratory barrier injury and severe allergic inflammation in mice

**DOI:** 10.1038/s41598-018-29689-9

**Published:** 2018-07-26

**Authors:** Thomas Secher, Isabelle Maillet, Claire Mackowiak, Jessica Le Bérichel, Amandine Philippeau, Corinne Panek, Michèle Boury, Eric Oswald, Abdelhadi Saoudi, Francois Erard, Marc Le Bert, Valérie Quesniaux, Aurélie Couturier-Maillard, Bernhard Ryffel

**Affiliations:** 1 IRSD, Université de Toulouse, INSERM, INRA, ENVT, UPS, Toulouse, France; 2 CNRS, UMR7355, Experimental and Molecular Immunology and Neurogenetics, Orleans, France; 3 CHU Toulouse, Hôpital Purpan, Service de Bactériologie-Hygiène, Toulouse, France; 4 Centre de Physiopathologie de Toulouse Purpan (CPTP), Université de Toulouse, UPS, Inserm, CNRS, Toulouse, France; 5 University of Orleans, Orleans, France; 6 University of Cape Town, IDM, Cape Town, Republic of South Africa; 7 Present Address: INSERM, UMR 1100, Research Center for Respiratory Diseases, and University of Tours, Tours, France

## Abstract

Allergic asthma is characterized by a strong Th2 and Th17 response with inflammatory cell recruitment, airways hyperreactivity and structural changes in the lung. The protease allergen papain disrupts the airway epithelium triggering a rapid eosinophilic inflammation by innate lymphoid cell type 2 (ILC2) activation, leading to a Th2 immune response. Here we asked whether the daily oral administrations of the probiotic *Escherichia coli* strain Nissle 1917 (ECN) might affect the outcome of the papain protease induced allergic lung inflammation in BL6 mice. We find that ECN gavage significantly prevented the severe allergic response induced by repeated papain challenges and reduced lung inflammatory cell recruitment, Th2 and Th17 response and respiratory epithelial barrier disruption with emphysema and airway hyperreactivity. In conclusion, ECN administration attenuated severe protease induced allergic inflammation, which may be beneficial to prevent allergic asthma.

## Introduction

Allergic asthma is one of the most common chronic respiratory diseases with a significant impact on public health^1,2^. In recent years, the incidence of allergic asthma in developed countries has dramatically increased and it is predicted that the number of affected people worldwide will increase by 100 million by 2025^3^. Risk alleles have been identified for the development of asthma^4^ but the rapidity of its increased incidence does not support solely a genetic basis and suggest the involvement of environmental factors. Long-term observations support the notion that urban life is associated with increased prevalence of chronic immunological disorders including asthma incidence as compared to children living in farms^5^. Early in life microbial exposure might modulate allergic disorders^6^. In addition, such favorable socioeconomic factors, like enriched dietary habits or increased level of hygiene are presumably important factors for a considerable shift in the gut microbiota and increased asthma susceptibility. Epidemiological and clinical studies indicate an association between alteration of intestinal microbial communities and increased incidence of allergic asthma^7^. Several studies revealed changes in gut microbiota composition in adults suffering from allergic diseases at distant body sites (eczema, rhinitis, asthma)^8,9^, which precede the development of allergic diseases^10,11^. Gut bacteria outnumber the human body cells and the microbiome encode approximately 100 times more genes than the human genome^12^. This impressive genetic capacity contribute to essential functions for the host including nutrients supply like short-chain fatty acids (SCFAs)^13,14^, vitamins and hormones^15^, energy balance^16–18^, metabolic signaling^19^, resistance to pathogens colonization^20–22^ and has a key role in promoting the postnatal maturation of the intestinal mucosal barrier^23–25^.

Asthma etiology is complex, but exposure to allergens or air pollution, are clearly important factors for the pathogenesis^5^. Sensitization to allergen is one of the first steps involved in asthma. Various allergens, including house dust mite (HDM), fungi, cockroach and pollen have proteolytic activities^26^. Protease properties of allergens cause injury of the airway epithelium with increased permeability, airway remodeling, type 2 cytokine and chemokine production and cell recruitment^27^. Papain, a cysteine protease, induces a type 2 response characterized by interleukin (IL)-5 and IL-13 production, mediated by an IL-2-dependent IL-9 production^28^ and specific IgE production^29,30^. There is evidence that the commensal microflora is critical in the maintenance of systemic immune tolerance, which is instrumental in protecting against allergic asthma. *Escherichia coli* strain Nissle 1917 (Mutaflor®, ECN) is successfully used for the treatment of intestinal inflammation, especially in patients suffering from ulcerative colitis^31^. In the present study, we investigated the impact of the colonization by ECN on the allergic lung inflammatory response induced by single or repeated challenges to the protease allergen papain. We show here that chronic ECN administration reduces severe allergic lung inflammation, improves the respiratory epithelial barrier function and modulates emphysema in response to repeated papain challenges.

## Results

### ECN colonization has a dual effect in acute papain-induced lung inflammation

**Figure 1:**
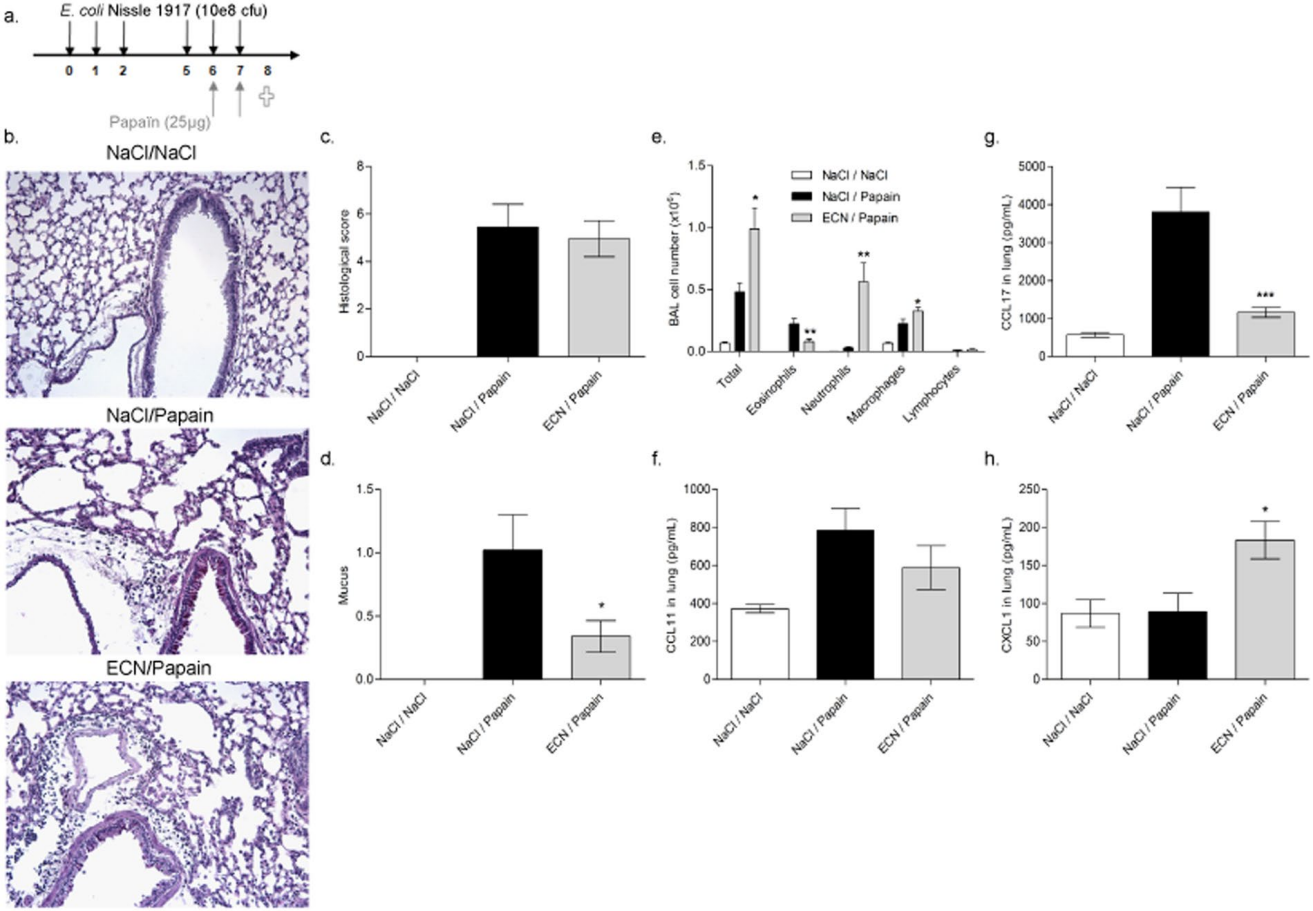
ECN colonization as a dual effect in acute papain-induced lung inflammation. (**a**) Experimental settings of acute papaïn-induced lung inflammation and ECN treatment. (**b**) Lung tissues were histologically examined 24 h after the last papaïn challenge. Lung sections stained with HE from controls (NaCl/NaCl), papaïn (NaCl/Papaïn) and ECN (ECN/Papaïn)-treated mice are represented. (**c**) Histological score of lung inflammation infiltration was performed on paraffin embedded section after HE staining. (**d**) Histological score of lung mucus production was performed on paraffin embedded section after PAS staining. (**e**) Total cells and differential cell count of eosinophils, neutrophils, lymphocytes and macrophages were determined in BALF by numeration of MGG stained cytospin. Lung homogenate level of (F) CCL11, (**g**) CCL17 and (**h**) CXCL1 were measured by ELISA. Data are expressed as mean + SEM from a single experiment representative of 2 experiments with n = 5 mice per group. The parametric one-way or two-way ANOVA test with multiple Bonferroni’s comparison test was used. *, ** and *** refer to *P* < 0.05, *P* < 0.01 and *P* < 0.001, respectively.

To study the impact of the administration of the ECN strain on the development of allergic inflammation, we compared the susceptibility ECN treated mice to acute papain-induced lung inflammation in comparison to non-treated controls according to the protocol shown in Fig. 1a. ECN was administered by gavage over 6 days (10^8^ cfu of live ECN/day) then the mice were challenged twice by intranasal instillation (i.n.) of the protease allergen papain (25 µg on day 7 and 8 and the inflammatory response was analyzed 24 h later as described before^32^. Microscopic examinations of the lungs revealed focal inflammatory cell infiltration around bronchi, capillaries and in alveoli, as well as mucus hypersecretion (Fig. 1b). The lung inflammation as assessed by a semi-quantitative score of microscopic lesions was not reduced in ECN fed mice (Fig. 1b,c), except for the production of mucus (Fig. 1d).

Papain-induced lung inflammation is associated with enhanced cell recruitment in the lung, involving especially eosinophils^32^. Cell recruitment into the broncho-alveolar lavage fluid (BALF) was modulated with increased total cells, especially neutrophils upon ECN treatment as compared to control mice (Fig. 1e) with increased myeloperoxidase (MPO) (Supplementary Figure 1) and neutrophil chemoattractant CXCL1 levels (Fig. 1h). By contrast, the recruitment of eosinophils in the BALF was significantly decreased in ECN-treated animals as compared to papain controls (Fig. 1e). This was correlated with a lowered production of CCL17 (Fig. 1g) while CCL11 levels was not modified (Fig. 1f).

Interestingly, mice treated with a non-probiotic K12 *E*. *coli* strain MG1655 and tested in the acute papain model (Supplementary Figure 2A) develop a similar lung neutrophilia as compared to ECN-treated animals (Supplementary Figure 2B–D), suggesting that this effect is probably mediated an E. coli genus dependent molecular determinant. On the contrary, MG1655 treatment has no protective effect on eosinophilia as observed with cell count and chemokine production (Supplementary Figure 2B,E,F). Taken together, these results suggest that gut colonization by ECN may modulate lung inflammation by enhancing neutrophil, but importantly reducing eosinophil cell recruitment in BALF and tissue. This data motivated studies in a chronic model of lung allergic inflammation.

### Chronic lung inflammation induced by repeated papain challenges is attenuated by ECN administration

**Figure 2:**
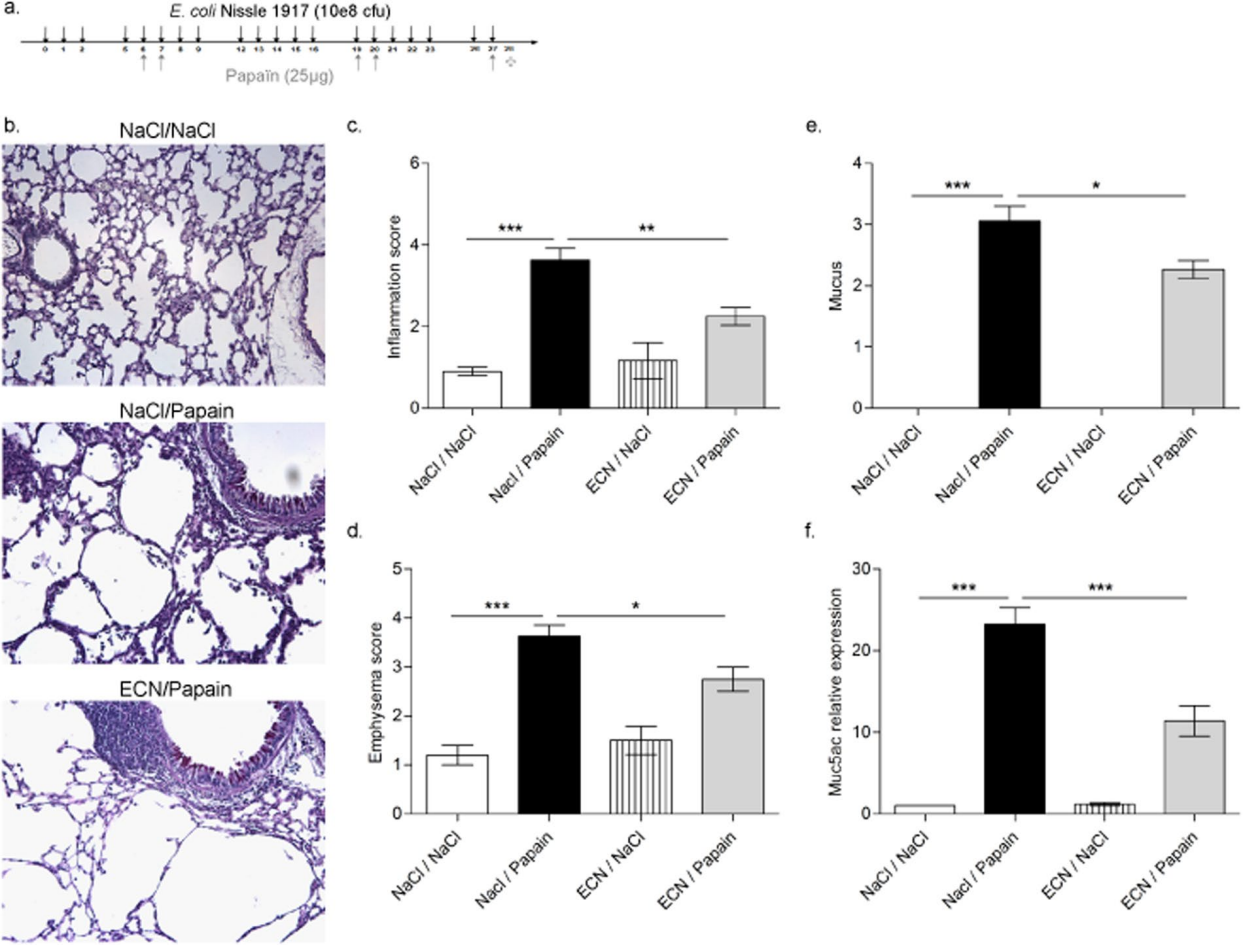
Repeated papain challenges causing severe lung inflammation is attenuated by ECN administration. (**a**) Experimental settings of chronic papaïn-induced lung inflammation and ECN treatment. (**b**) Lung tissues were histologically examined 24 h after the last papaïn challenge. Lung sections stained with HE from controls (NaCl/NaCl), papaïn (NaCl/Papaïn) and ECN (ECN/Papaïn)-treated mice are represented. (**c**) Histological score of lung inflammation infiltration was performed on paraffin embedded section after HE staining. (**d**) Histological score of airway remodeling was performed on paraffin embedded section after HE staining. (**e**) Histological score of lung mucus production was performed on paraffin embedded section after PAS staining. (**f**) *Muc5ac* relative gene expression levels in lung tissues was measured by qPCR. Data are expressed as mean + SEM from a single experiment representative of 2 experiments with n = 5 mice per group. The parametric one-way or two-way ANOVA test with multiple Bonferroni’s comparison test was used. *, ** and *** refer to *P* < 0.05, *P* < 0.01 and *P* < 0.001, respectively.

To determine whether ECN modulates chronic airway inflammation induced by a protease allergen papain, BL6 mice were immunized with papain (25 µg on days 6, 7 by intranasal route), followed by two intranasal challenges at day 20 and 25 (25 µg). Control mice received vehicle (NaCl). In addition, mice were orally administered with 10^8^ cfu of live ECN (Fig. 2a). 24 h after the last papain challenge, the mice were sacrificed and the extent of the lung inflammation was assessed. Histological analysis revealed a prominent lung inflammation characterized by perivascular, peribronchial and alveolar infiltration of eosinophils, neutrophils and air space enlargement with epithelial damage and disruption of alveolar septa, a hallmark of emphysema upon papain challenge (Fig. 2b,c). ECN-treated mice largely prevented lung inflammation, epithelial injury and emphysema (Fig. 2b–d). Finally, the extensive goblet cell hyperplasia and mucus production observed in primed/challenged mice was lowered in ECN probiotic treated mice (Fig. 2b,e). Diminished mucus expression was confirmed at the mRNA level for Muc5ac in lung (Fig. 2f). Interestingly, mice treated with *E*. *coli* strain MG1655 and tested in the chronic papain model develop a similar lung inflammation as compared to untreated animals, as revealed by the histological analysis (Supplementary Figure 3A–E), suggesting that the protective effect observed with ECN is due to intrinsic probiotic properties rather than a non-specific effect due to daily gavage *E*. *coli* species on the gut microbiota. The absence of protection with MG1655 is unlikely related to the lack of gut colonization, as we quantified equivalent *Enterobacteria* and *E*. *coli* colony counts in both ECN- and MG1655-treated animals along the treatment (Supplementary Figure 4).

### ECN-treated mice develop reduced airway eosinophilia and Th2-driven airway inflammation upon papain chronic challenges

**Figure 3:**
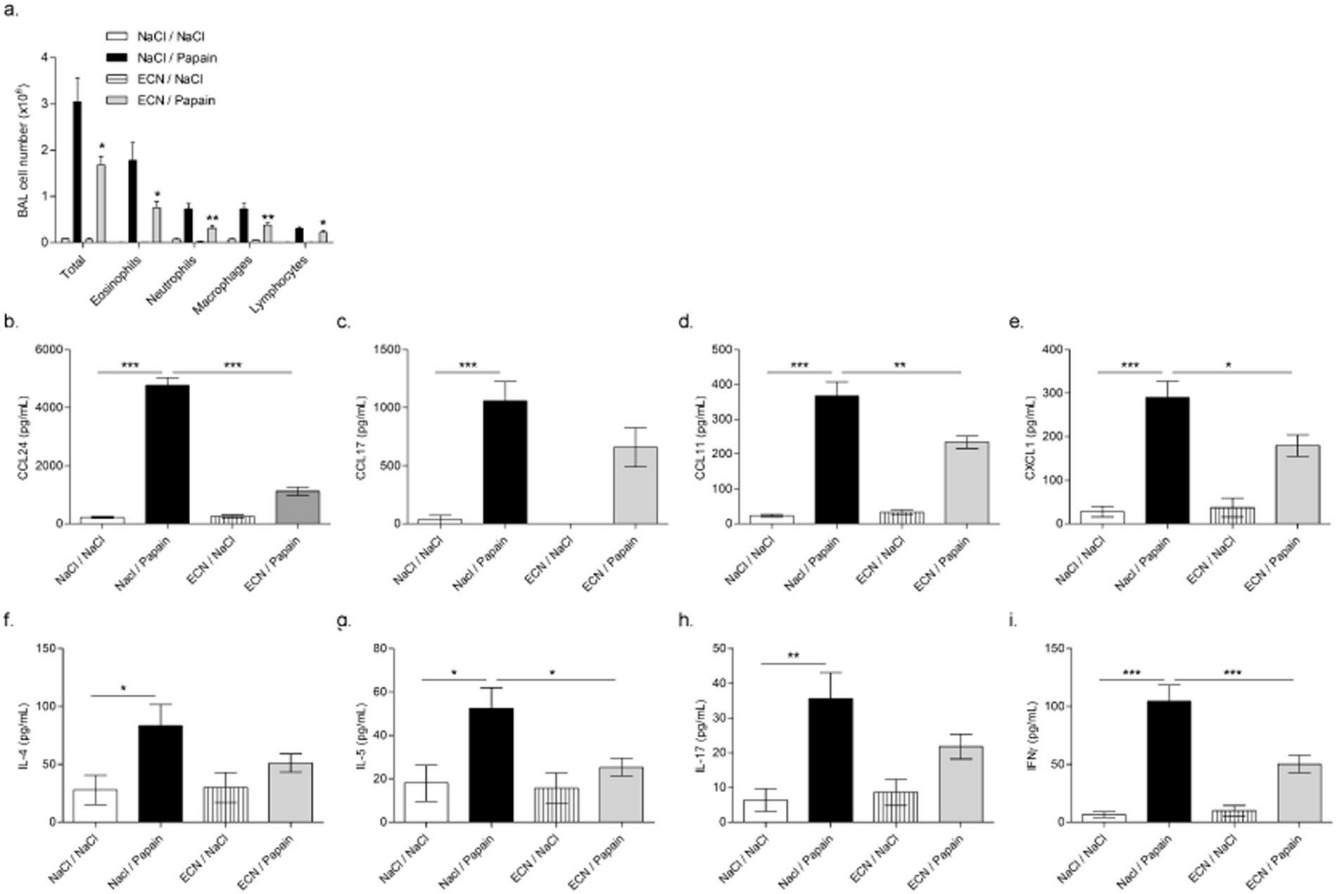
ECN-treated mice develop reduced airway eosinophilia and Th2-driven airway inflammation upon papaïn chronic challenges. (**a**) Total cells and differential cell count of eosinophils, neutrophils, lymphocytes and macrophages were determined in BALF by numeration of MGG stained cytospin. Lung homogenate level of (**b**) CCL24, (C) CCL17, (D) CCL11, (**e**) CXCL1, (**f**) IL-4, (**g**) IL-5, (**h**) IL-17 and (**i**) IFNγ were measured by ELISA. Data are expressed as mean + SEM from a single experiment representative of 2 experiments with n = 5 mice per group. The parametric one-way or two-way ANOVA test with multiple Bonferroni’s comparison test was used. *, ** and *** refer to *P* < 0.05, *P* < 0.01 and *P* < 0.001, respectively.

Papain-induced chronic inflammation is characterized by a type 2 inflammatory response^28^. To determine whether ECN inhibited inflammatory cell recruitment, BALF cell counts were assessed for cell phenotyping. Saline sensitized and challenged mice present negligible leukocyte numbers in BALF, whereas papain-treated mice presented a dramatic increase of total cells, eosinophils and fewer neutrophils and macrophages (Fig. 3a). By contrast, ECN-treated mice had ~1.5 less total BALF cell counts with a 2-fold reduction in eosinophils, neutrophils and macrophages. This was consistent with significant lower levels of eosinophils attracting chemokines CCL24 and CCL11 (Fig. 3b,d), EPO levels (Supplementary Figure 5) and neutrophils/monocytes chemoattractant CXCL1 (Fig. 3e), while CCL17 was unchanged in the lungs of ECN-treated mice as compared to controls. Moreover, Th2 cytokines such as IL-5 and to a lesser extent IL4 were significantly reduced in the lung of ECN-treated mice as compared to papain controls (Fig. 3f,g). The production of IFNγ was reduced, while IL17A level was unchanged in ECN probiotic-treated mice (Fig. 3h,i).

Taking together, these data indicate that ECN gut colonization reduces papain induced Th2 immune response.

### Papain-induced airways hyperreactivity and respiratory barrier injury is attenuated

**Figure 4:**
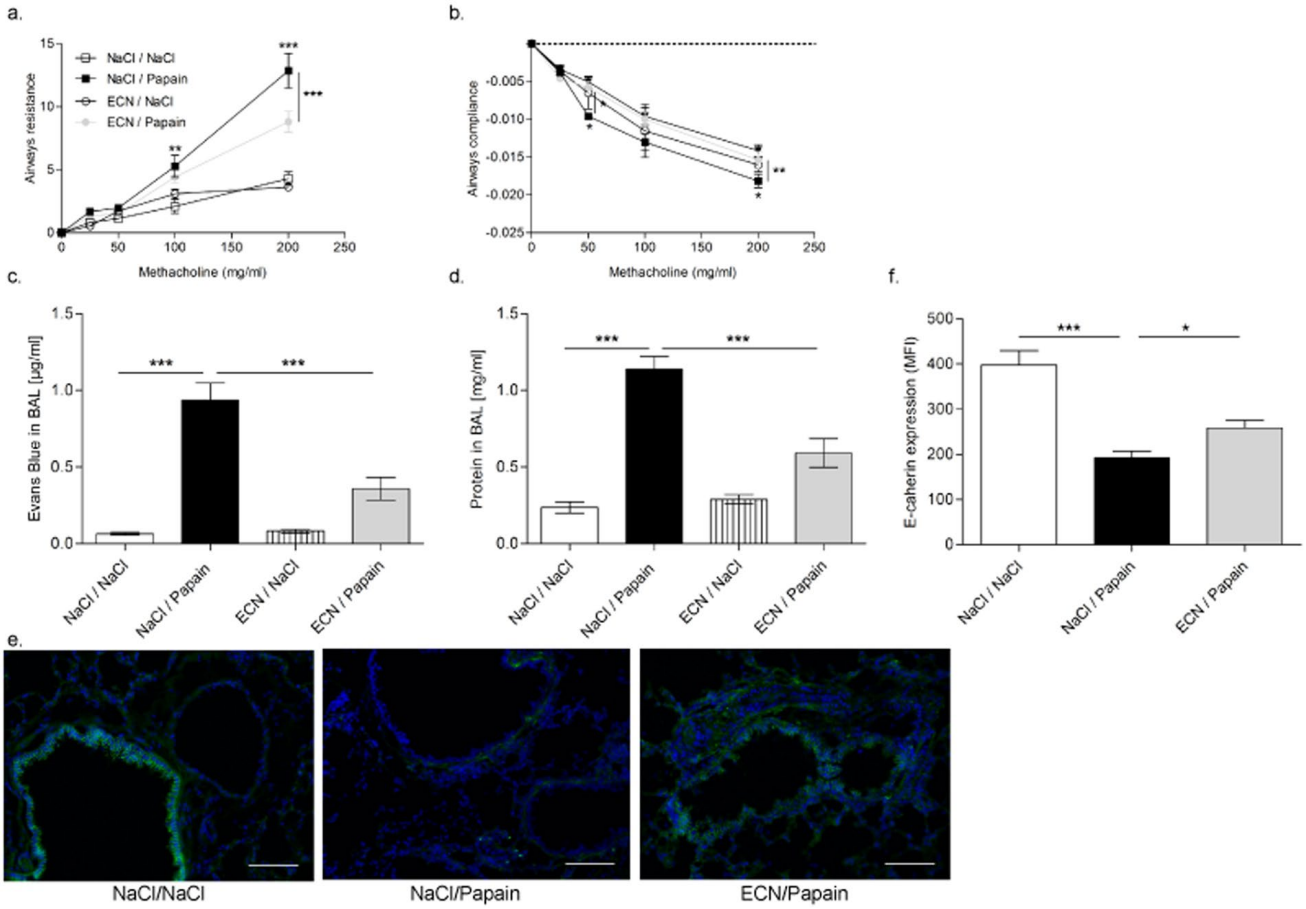
Papaïn-induced pulmonary dysfunction is attenuated by ECN. (**a**) Airway hyper-responsiveness to increasing doses of methacholine (Mch; 0−200 mg/ml) was measured by recording changes in lung resistance and (**b**) airway compliance. The pulmonary epithelial integrity was assessed by the leak of (**c**) Evans blue and (**d**) total protein in BAL. (**e**) Immunofluorescent staining for E-cadherin (green) on lung cryosections. (**f**) Quantitative evaluation of E-cadherin expression on lung sections. Data are expressed as mean + SEM from a single experiment representative of 2 experiments with n = 5 mice per group. The parametric one-way or two-way ANOVA test with multiple Bonferroni’s comparison test was used. *, ** and *** refer to *P* < 0.05, *P* < 0.01 and *P* < 0.001, respectively.

A hallmark of allergic lung inflammation is airways hyperreactivity (AHR), which is due functional changes of the respiratory barrier. AHR was assessed by invasive plethysmography in untreated and ECN-treated mice upon chronic papain exposure. Airway resistance and compliance in response to methacholine as a measure of AHR and were increased upon papain challenge. ECN administration reduced airway resistance and compliance indicating a significant amelioration of the lung function (Fig. 4a,b).

The protease papain induces inflammation and injury of the lung epithelium and capillaries with increased vascular permeability. The probiotic ECN has the ability to strengthen the epithelial barrier^33^. We used Evans Blue (EB), which binds to serum albumin, as a tracer of the capillary leak of macromolecules from the circulation into the BALF. Our data reveal that ECN treatment reduced the acute lung capillary/epithelial leak of intravenous administered EB upon papain exposure (Fig. 4c). Furthermore, total protein in BALF was also reduced (Fig. 4d). To get further insights into the role of ECN in the improvement of lung epithelial barrier function during allergic asthma, lung histological sections were analyzed for the expression of E-cadherin, a critical component of the epithelial barrier, which is crucial in the maintenance of the immunologic tolerance during airway allergic sensitization^34^. Immunofluorescence analysis revealed reduced E-cadherin expression concomitant with epithelial cell injury upon papain exposure, while ECN feeding attenuated the reduction of E-cadherin expression (Fig. 4e), which was confirmed by a semi-quantitative assessment of E-cadherin immunostaining (Fig. 4f).

Therefore ECN colonization attenuated papain protease induced allergic lung inflammation with reduced Th2 response and airways hyperreactivity. Importantly the protease induced injury of the alveolar septae reflected by emphysema and of the respiratory barrier were significantly diminished by the probiotic strain ECN.

### ECN-treated mice has reduced Th2 lymphocytes and ILC2 activation upon papain chronic challenges

**Figure 5:**
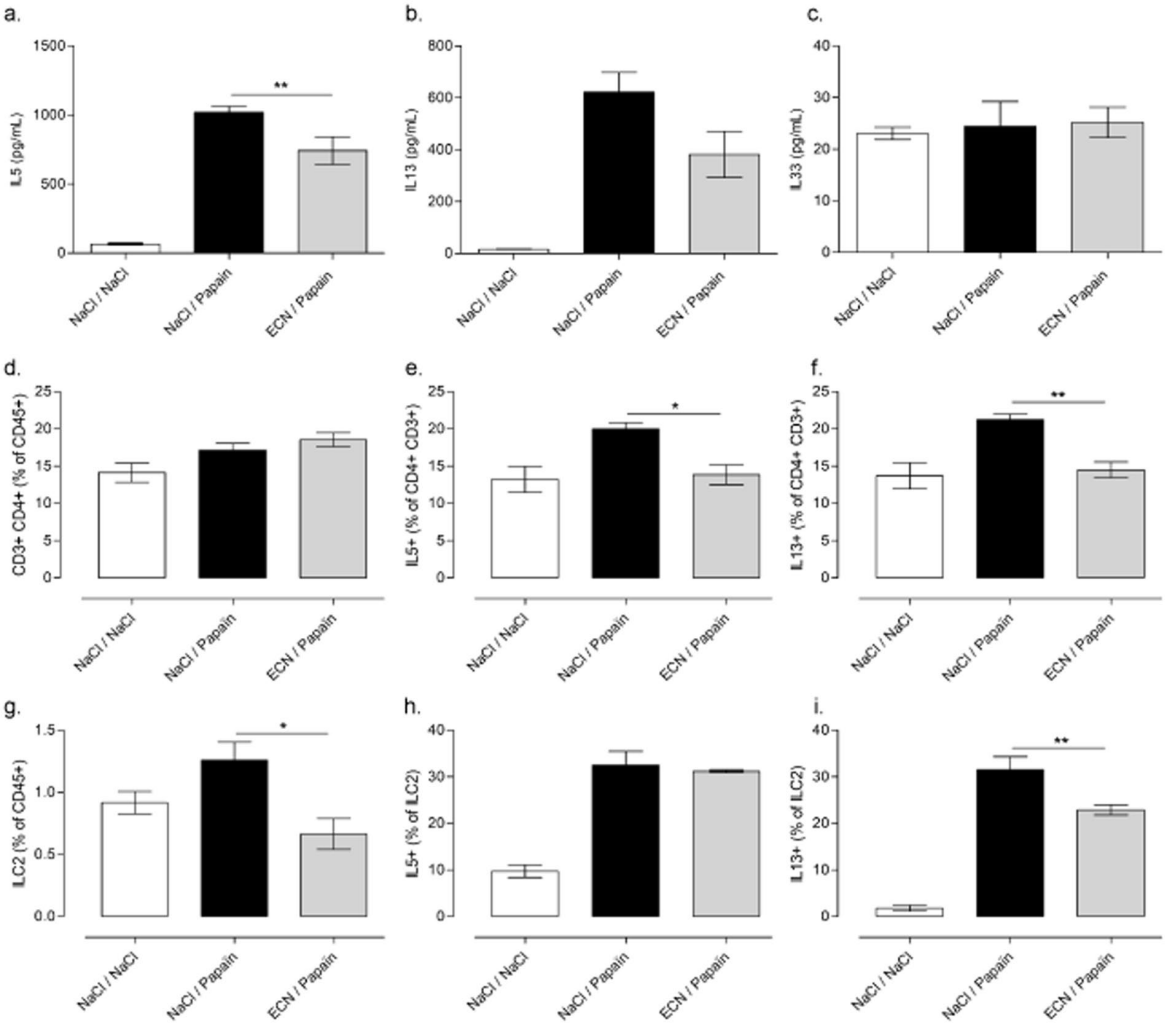
ECN-treated mice has reduced Th2 lymphocytes and ILC2 activation upon papain chronic challenges. IL-5 (**a**), IL-13 (**b**) and IL-33 (**c**) levels after lung mononuclear cell restimulation with papaïn for 72 h. Frequency of CD3+ CD4+ lymphocytes (**d**) producing IL-5 (**e**) or IL-13 (**f**) are shown. Frequency of ILC2 (**g**) producing IL-5 (**h**) or IL-13 (**i**) are shown. Data are expressed as mean + SEM from a single experiment with n = 5 mice per group. The parametric one-way or two-way ANOVA test with multiple Bonferroni’s comparison test was used. * and ** refer to *P* < 0.05 and *P* < 0.01, respectively.

Th2 lymphocytes and ILC2 accumulate in lungs after papaïn exposure and produce IL-5 and IL-13^35^. We determine the relative contribution of ECN on Th2 and ILC2 activation 24 h after the last allergen challenge. Lung cells were restimulated by papain and the production of cytokines was analyzed. IL-5 (Fig. 5a) and to a lesser extent IL-13 (Fig. 5b) was significantly reduced upon ECN treatment while IL-33 levels remain unchanged (Fig. 5c). Total Th2 and ILC2 producing IL-5 and IL-13 were analyzed by flow cytometry (Supplementary Figures 6 and 7). The frequency of CD3+ CD4+ IL5+ or IL13+ cells were significantly reduced in ECN-treated mice as compared to untreated controls (Fig. 5d–f). This was associated with a similar decrease of ILC2+ and ILC2+ IL13+ (Fig. 5g–i). These data indicate that ECN was able to dampen Th2 and ILC2 activation and the production of the prototypal pro-allergenic IL-5 and IL-13.

## Discussion

Allergic asthma is a major health issue with increasing incidence especially in developed countries with an epidemic feature^36^. Asthma etiology is complex including both genetic and environmental factors, such as exposure to allergens and/or air pollution, are important for the pathogenesis^5^. Data regarding the use of probiotics in the prevention of allergic diseases and asthma are conflicting^37^. Several different bacterial strains or combinations have been used in clinical trials to assess protective effects in the context of allergic asthma with significant reduction of both incidence and severity of allergic diseases^38^ which were not confirmed by others^39^. A meta-analysis concluded that probiotic are not efficient for the prevention of allergy^40^. This discrepancy may be related to the dose and duration of probiotic administration, immunomodulatory differences^41^ among strains, mostly *Lactobacillus* or *Bifidobacterium* probiotics^42^. Here we evaluated the probiotic potential of the Gram negative ECN to prevent allergic lung inflammatory allergic response induced by the protease papain. ECN drastically reduced the severity of chronic lung inflammation through the modulation of the Th2 inflammatory response, injury of the respiratory barrier and airways hyperreactivity. The beneficial effects of ECN has been demonstrated before in intestinal inflammatory disorders, especially in ulcerative colitis^43^. Two previous studies investigated ECN in experimental asthma. Bickert *et al*. using the inert protein allergen OVA observed a protection upon oral administration of ECN, but no inhibition of the Th2 immune response^44^. Adam *et al*. evaluated the prophylactic potential of ECN on recombinant house mite antigen Derp1 as mucosal antigen. ECN strongly reduced the antigen specific humoral response^45^. Here, using oral prophylactic administration of ECN we demonstrate for the first time a reduction of papain-induced lung inflammation and amelioration of AHR. In contrast, mice administered K12 *E*. *coli* strain MG1655 were as sensitive to lung inflammation as untreated papain challenged mice suggesting that the genetic background of the strain is of particular importance and determines its ability to act as a probiotic. Nevertheless, we observed that both *E*. *coli* strains has the ability to induce a potent lung neutrophilia. These results are in line with several papers demonstrating that ECN capsule antigen K5 was an important contributor the recruitment of neutrophil^46,47^. More generally, it has also been suggested that the presence of capsular antigen may induce an increased influx of pulmonary neutrophils^48,49^. The mechanisms by which capsular antigen modulate neutrophil response are not completely understood but may include direct effect such an upregulation of shed bacterial formylmethionyl-leucyl-phenylalanine^50^, a potent neutrophil chemotactic factor; or indirect by modulating the host’s generation of chemokines, including CXCL1 or IL-8 which was observed upon ECN or MG1655 treatment.

One of the best-characterized features contributing to the effectiveness of ECN is its ability to strengthen the epithelial barrier function^51^. This probiotic property of ECN has been extensively demonstrated in the context of intestinal inflammatory diseases. Asthma is often associated with mucosal barrier dysfunction^52^. We found that respiratory barrier dysfunction due to papain-induced inflammation and injury is alleviated by ECN with reduced protein leak and upregulation of E-cadherin. Recent studies suggests that this adhesion molecule contributes to the structural and immunological function of the airway epithelium, acting as a rheostat through the regulation of epithelial junctions and production of pro-inflammatory mediators^34^. Alterations of the airway epithelium enhance both allergic sensitization and airway remodeling including goblet cell hyperplasia, mucus hyperproduction and subepithelial fibrosis^53^ thus contributing to severe airways hyperreactivity. ECN conferred a significant reduction of inflammatory cell recruitment in BALF, lung tissue inflammation and disruption of alveolar septa with emphysema.

Airway epithelial cells participate in the innate immune response of the lung and have barrier function. Barrier dysfunction favors the access of noxious or immunogenic protein or chemicals to the mucosa-associated lymphoid tissues. Thus, regulation of airway epithelial barrier function is an important checkpoint of the immune response during asthma^54^. In the present study, we show that ECN treatment affects a prevalent Th2 response known for papain induced lung inflammation^28^. We observed a significant reduction of eosinophils and eosinophil-related chemokines/cytokines associated with diminished recruitment of neutrophils and CXCL1 and IFN-γ levels. The data are consistent with previous studies showing that colonization by ECN lead to a modification of the cytokines repertoire^55,56^. In addition, we show for the first time that ECN treatment reduce Th2 CD4+ lymphocytes as well as ILC2 activation, resulting in decreased IL-5 and IL-13 production. The latter population is known to precede Th2 activation which is the cardinal feature of allergic asthma, culminating in airway hyperresponsiveness and Th2 cytokines and chemokines. In this setting, we investigated IL-33, which is known to be involved in ILC2 activation^35^ but we did not find any difference upon ECN treatment, which was also the case in another reduced allergic asthma condition^57^.

The molecular rationale behind the immunomodulatory properties of ECN has not yet been elucidated and is under investigation^58^. The beneficial effect of ECN could rely on the improvement of the intestinal barrier function and the resulting prevention of a continuous stimulation of the host innate immune system by the gut components. Indeed, we have recently demonstrated that ECN was able to prevent CNS inflammation through the improvement of the intestinal permeability^59^ showing that modulation of the gut microbiota with ECN exerts remote immunological imprinting. ECN genome encodes the production of specialized molecules that may modulate immune functions^60–62^. The intestinal mucosa represents an interface between bacterial-derived metabolites and mucosal immune processes that will influence immunological processes on the host systemically^63^.

In conclusion, our findings indicate that ECN is able to prevent papain-induced lung inflammation after high dose *per os* administration supporting a gut-lung mucosal communication^64^. In addition, our results suggest that the prevention of the respiratory barrier dysfunction by probiotic treatment may be important to control allergic lung inflammation. Therefore, ECN might be considered as a valuable prophylactic or diet supplement to prevent allergic asthma.

## Methods

### Mice

C57BL/6 (B6) mice were bred in our specific pathogen free animal facility at TAAM-CNRS, Orleans, France (agreement D-45-234-6 delivered on March, 10 of 2014). Mice were maintained in a temperature-controlled (23 °C) facility with a strict 12 h light/dark cycle and were given free access to food and water. The experiments were performed with female mice aged 8–10 weeks using 5 mice per group, and the experiments were repeated at least twice. All animal experimental protocols were carried out in accordance with the French ethical and animal experiments regulations (see Charte Nationale, Code Rural R 214-122, 214-124 and European Union Directive 86/609/EEC) and were approved by the “Ethics Committee for Animal Experimentation of CNRS Campus Orleans” (CCO), registered (N°3) by the French National Committee of Ethical Reflexion for Animal Experimentation (CLE CCO 2013-1006).

### Bacterial preparation, growth conditions and administration

The strains used in this study are the probiotic *Escherichia coli* Nissle 1917 (ECN) and the archetypal K12 *E*. *coli* strain MG1655. Both strains were engineered to exhibit a mutation in the *rpsL* gene, which is known to confer resistance to streptomycin^62^. Before oral administrations, ECN and MG1655 strains were grown for 6 h in LB broth supplemented with streptomycin (50 µg/mL) at 37 °C with shaking. This culture was diluted 1:100 in LB broth without antibiotics and cultured overnight at 37 °C with shaking. Bacterial pellets from this overnight culture were diluted in sterile PBS to the concentration of 10^9^ colony forming units (cfu)/ml. Mice were treated by oral gavage with 10^8^ cfu of ECN or MG1655 in 100 µl of PBS or 100 µl of PBS as negative control.

### Papain-induced lung inflammation model in mice

Mice were anesthetized by an iv injection of ketamine/xylazine followed by an intranasal administration of 25 µg of papain (Calbiochem, Darmstadt, Germany) in 40 µL of saline solution. Mice were euthanized by CO_2_ inhalation 24 h after papain administration and BALF was collected. After a hearth perfusion with ISOTON II (Acid free balanced electrolyte solution Beckman Coulter, Krefeld, Germany) lung were collected and sampled for analyses.

### Broncho alveolar lavage (BAL)

BAL was performed by 4 lavages of lung with 500 µL of saline solution via a cannula introduced into mice trachea. BAL fluids were centrifuged at 400 g for 10 min at 4 °C, the supernatants were stored at −20 °C for ELISA analysis and pellets were recovered to prepare cytospin (Thermo scientific, Waltham, USA) glass slides followed by a Diff-Quik (Merz & Dade A.G., Dudingen, Switzerland) staining. Differential cell counts were performed with at least 400 cells.

### Pulmonary eosinophil peroxidase (EPO) activity

EPO activity was determined in order to estimate the recruitment of eosinophil counts in lung parenchyma as described^65^.

### Muc5ac expression

Total RNA was isolated from homogenized mouse lung using Tri Reagent (Sigma) and quantified by NanoDrop (Nd-1000). Reverse transcription was performed withSuperScript III Kit according to manufacturers’ instructions (Invitrogen). cDNA was subjected to quantitative PCR using primers for Muc5ac (sense 5′-CAGCCGAGAGGAGGGTTTGATCT-3′ and anti-sense 5′-AGTCTCTCTCCGCTCCTCTCA-3′; Sigma). Relative transcript expression of a gene is given as 2−ΔCt(ΔCt = Cttarget−Ctreference), and relative changes compared with control are 2−ΔΔCtvalues (ΔΔCt = ΔCttreated−ΔCtcontrol) {John, 2014 #340}.

### Enzyme-linked Immunosorbent assay (ELISA)

Homogenized lung or cell supernatant were tested for MPO, CXCL1, CCL24, CCL11, CCL17, IL-4, IL17A and IFNγ (R&D systems Abingdon, UK), IL-13, IL-5, IL-33 (eBiosciences, San-5, Diego, USA) using commercial ELISA kits according to the manufacturer’s instructions.

### Histology

The left lobe of lung was fixed in 4% buffered formaldehyde and paraffin embedded under standard conditions. Tissue sections (3 µm) were stained with PAS. Histological changes such as inflammation and emphysema were assessed by a semi-quantitative score from 0 to 5 for cell infiltration (with increasing severity) as described before^66^. The slides were examined by two blinded investigators with a Leica microscope (Leica, Germany).

### Determination of bronchial hyperresponsiveness (AHR)

For invasive measurement of dynamic resistance, mice were anesthetized with intra-peritoneal injection of solution containing ketamine (100 mg/kg, Merial) and xylasine (10 mg/kg, Bayer), paralyzed using D-tubocuranine (0.125%, Sigma), and intubated with an 18-gauge catheter. Respiratory frequency was set at 140 breaths per min with a tidal volume of 0.2 ml and a positive end-expiratory pressure of 2 ml H_2_O. Increasing concentrations of aerosolized methacholine (9.375, 18.75, 37.5, 75 and 150 mg/ml) were administered. Resistance was recorded using an invasive plethysmograph (Buxco, London, UK). Baseline resistance was restored before administering the subsequent doses of methacholine.

### E-cadherin immunofluorescence staining

Lungs were fixed for 3 days in 4% PFA and submerged in 20% sucrose for 1 week. Lungs were embedded in OCT (Tissue-Teck) and 10 µM sections were prepared with cryotome (Leica). Slides were incubated 30 min in citrate buffer at 80 °C, washed in TBS-Tween and then incubated overnight with mouse-anti-mouse-E-cadherin (1 µg/ml, ab76055, Abcam). After washing with slides were treated with 0,05% pontamin sky blue (Sigma) for 15 min and then incubated with secondary AF-546 goat anti-mouse antibody (Abcam) for 30 min at room temperature. After washing, slides were incubated with DAPI (Fisher Scientific) and mounted in fluoromount® (SouthernBiotech). Lung sections were observed on a fluorescence microscope Leica (Leica, CTR6000) at x200 magnification. The slides were analyzed and semi-quantitatively scored and the MFI was measured.

### Lung epithelial barrier function

Total protein in BAL fluid and Evans blue EB leak in BAL fluid was determined as described before^65^.

### Lung mononuclear cell isolation and stimulation

Lung mononuclear cells were isolated from mice 24 h after the last challenge as described previously^67^. Briefly the aorta and the inferior vena cava were sectioned and the lungs were perfused with 10 mL of saline. The lobes of the lungs were sliced into small cubes and then incubated for 45 min in 1 ml of RPMI 1640 solution and digested in 1,25 mg/ml of Liberase TL (Roche Diagnostics) and 1 mg/ml DNAse 1 (Sigma) during 1 h at 37 °C. Red blood cells were lysed with lysing buffer (BD Pharm Lyse^TM^ – BD Pharmingen). Isolated lung mononuclear single live cells were plated in round bottom 96-well plates (1 × 10^6^/ml) and restimulated 3 h at 37 °C with PMA (50 ng/mL) and ionomicyn (750 ng/mL) in the presence of Brefeldin A (1 μl/1 × 10^6^ cells, BD Biosciences) for intracellular flow cytometry analysis. Lung mononuclear cell (1 × 10^6^ cells) were restimulated with 25 µg of papain in RPMI and 10% SVF at 37 °C in a 96 well plate for 3 days. Supernatants were analyzed for the presence of IL-5, IL-13 and IL-33 by ELISA (invitrogen).

### Flow cytometry analysis on lung mononuclear cells

Lung mononuclear cells were stained with V450-conjugated anti-CD45 (clone 30F11), PerCp cy5.5-conjugated anti-CD3e (clone 145-2C11), FITC-conjugated anti-CD4 (clone RM4-5), PE-Cy7 -conjugated anti-ICOS (clone 7E.17G9), FITC-conjugated anti-ST2 (clone U29-93), PercP-Cy5.5 anti B220 (clone RA3-6B2), PercP-Cy5.5 anti FcεRI (clone MAR-1), PercP-Cy5.5 anti CD11b (clone M1/70), PercP-Cy5.5 anti Siglec-F (clone E50-2440) and Fixable Viability Dye eFluor™ 780 (eBioscience). After washing, cells were permeabilized for 20 min with cytofix/cytoperm kit (BD Biosciences) and stained with, eFluor 660-conjugated anti-IL13 (clone eBio13A, eBiosciences) and PE-conjugated anti-IL-5 (clone TC11-18H10.1). All antibodies used in this were from BD Biosciences, unless otherwise specified. Data were acquired using FACS Canto II flow cytometer and analyzed using Diva and FlowJo software.

### Statistical analysis

Data were analyzed using Prism version 5 (Graphpad Software, San Diego, USA). The parametric one-way ANOVA test with multiple Bonferroni’s comparison test was used. Values are expressed as mean ± SEM. Statistical significance was defined at a p-value < 0.05.

## Electronic supplementary material

Supplementary information
