# Supplementary material for: The probiotic strain Escherichia coli Nissle 1917 prevents papain-induced respiratory barrier injury and severe allergic inflammation in mice
Source: Sci Rep. 2018 Jul 26;8:11245. doi: 10.1038/s41598-018-29689-9 (PMC6062509; doi:10.1038/s41598-018-29689-9)
Supplement: Supplementary file 1 — Supplementary information [file 41598_2018_29689_MOESM1_ESM.docx]

**The probiotic strain *Escherichia coli* Nissle 1917 prevents papain-induced respiratory barrier injury and severe allergic inflammation in mice**

Thomas Secher, Isabelle Maillet, Claire Mackowiak, Jessica Le Bérichel, Amandine Philippeau, Corinne Panek, Michèle Boury, Eric Oswald, Abdelhadi Saoudi, Francois Erard, Marc Le Bert, Valérie Quesniaux, Aurélie Couturier-Maillard and Bernhard Ryffel

**Supplementary figures**
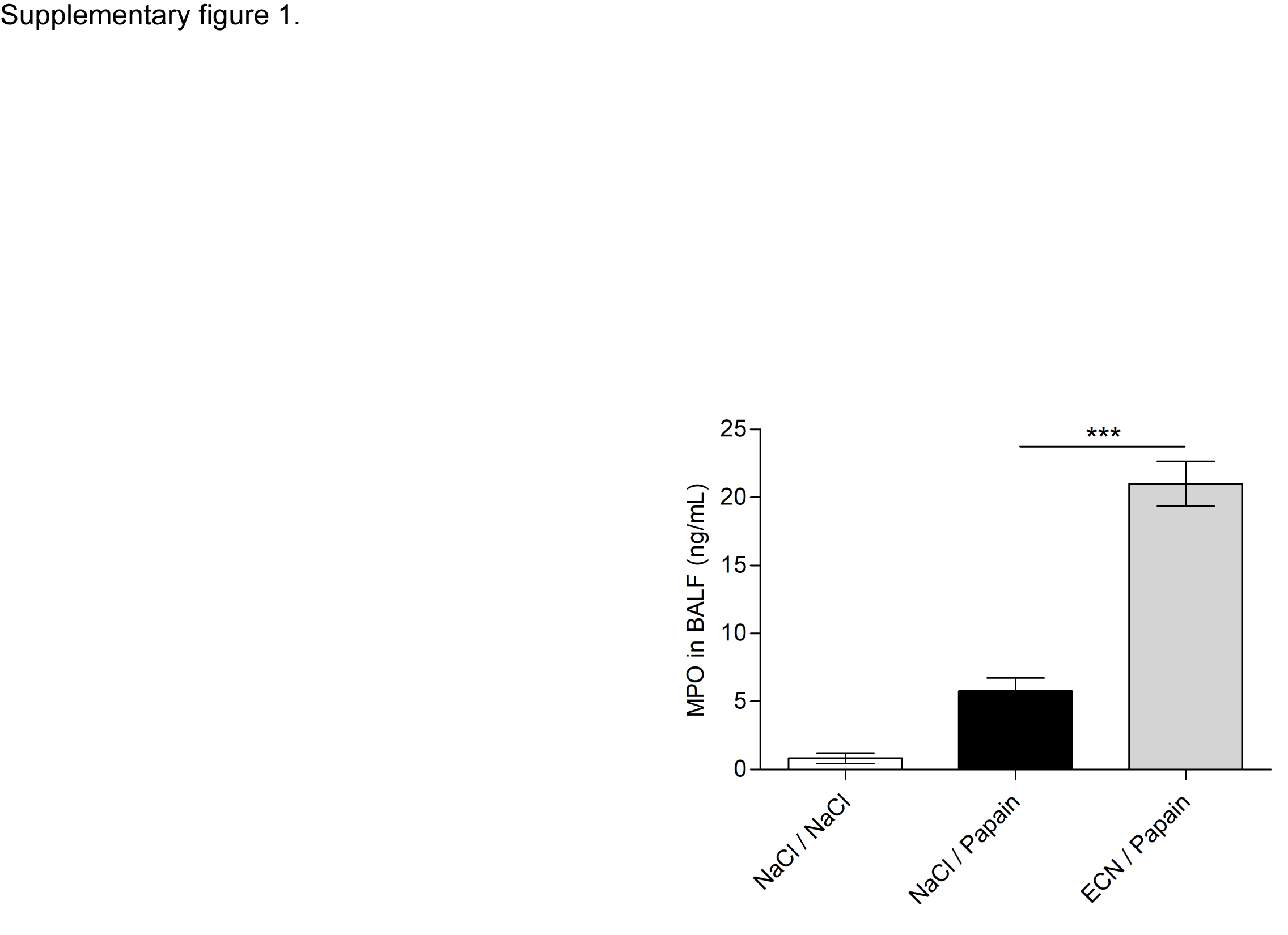


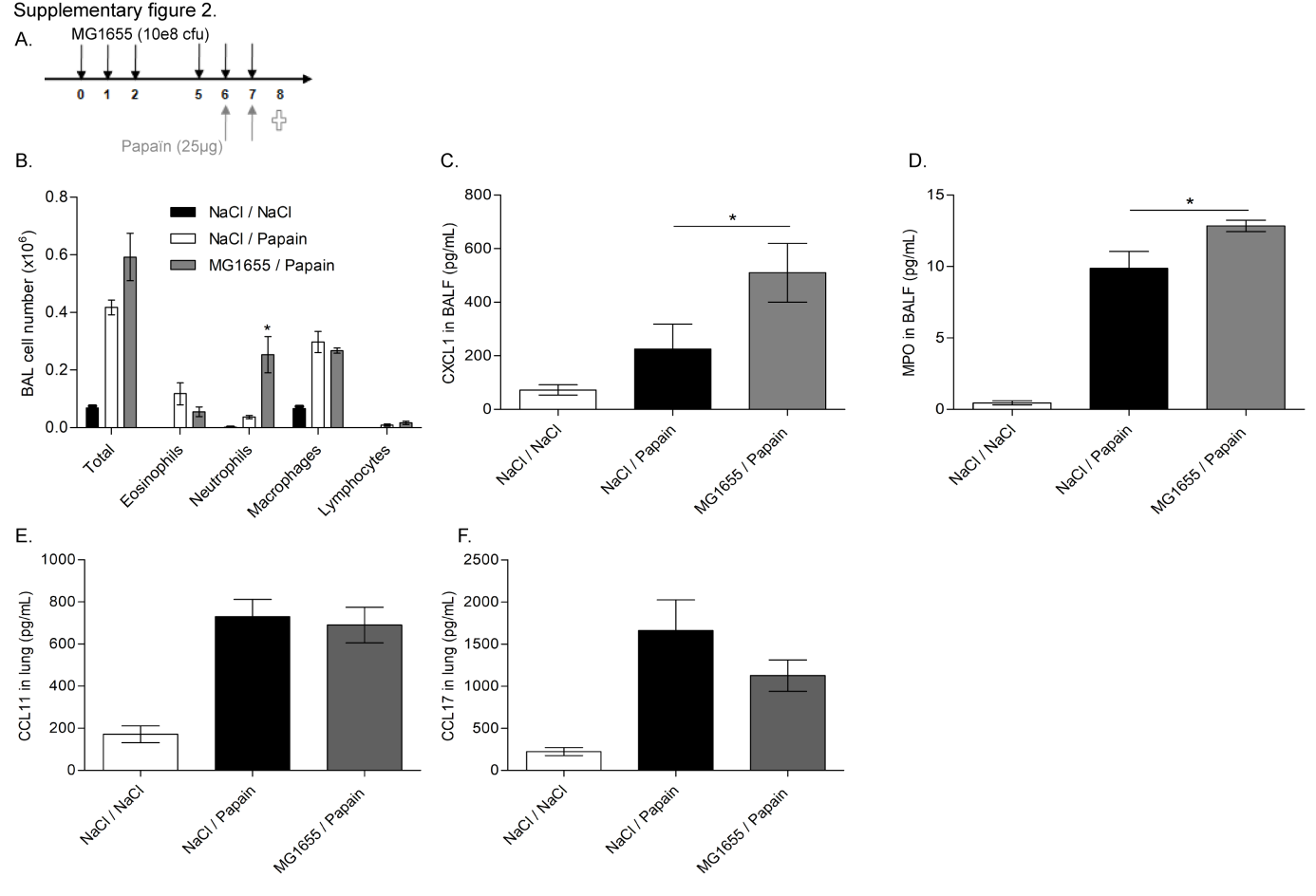


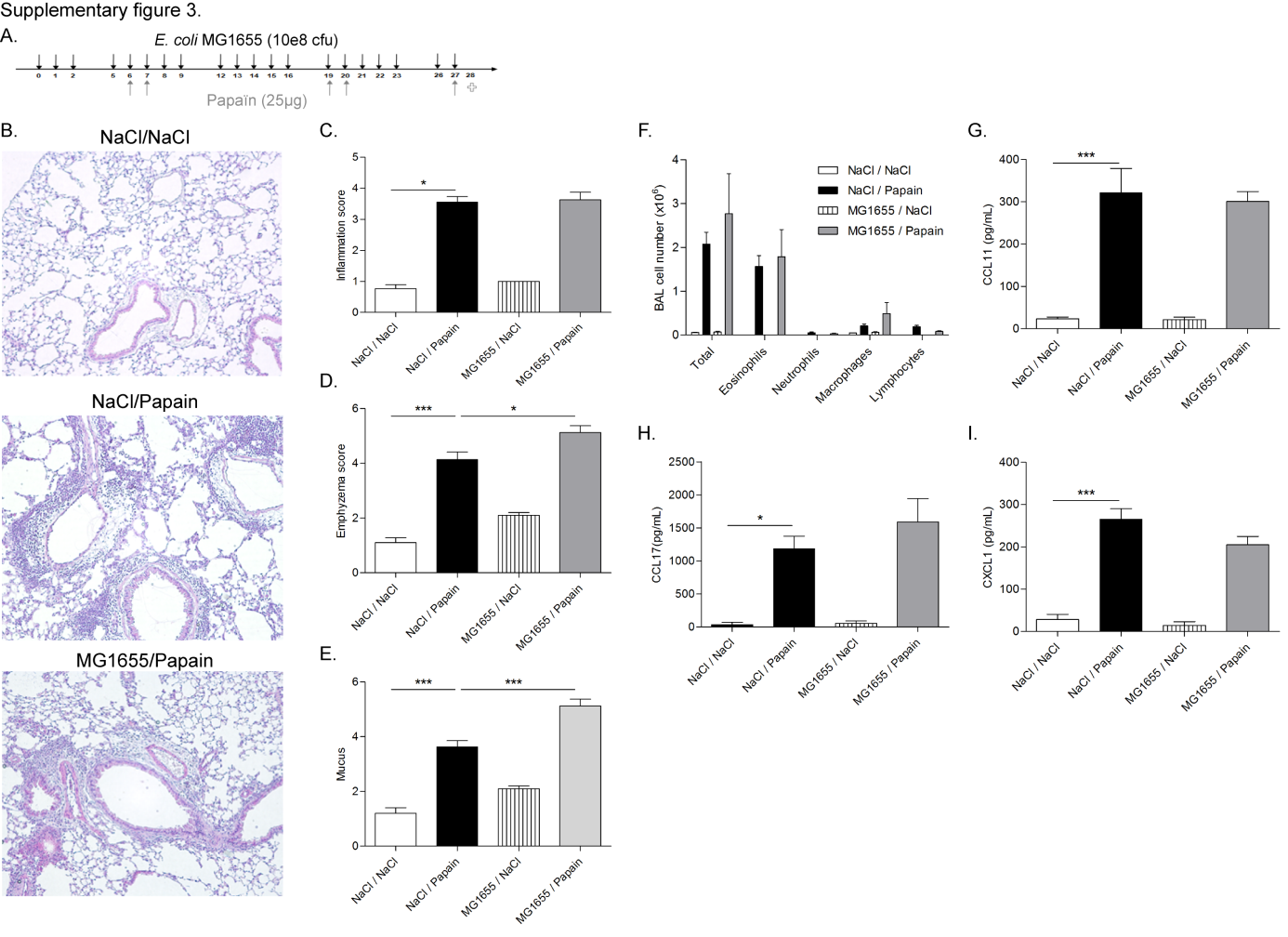


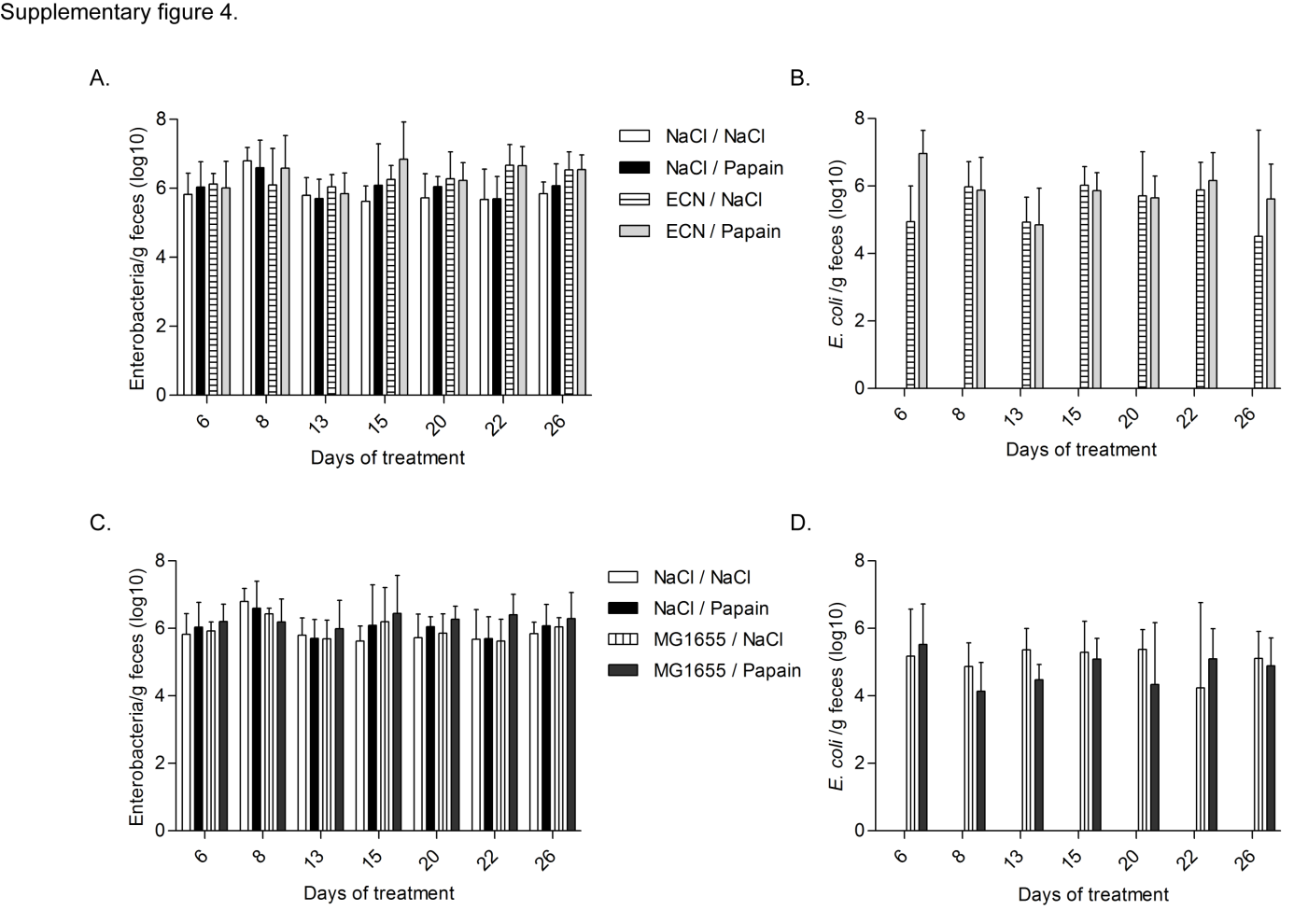


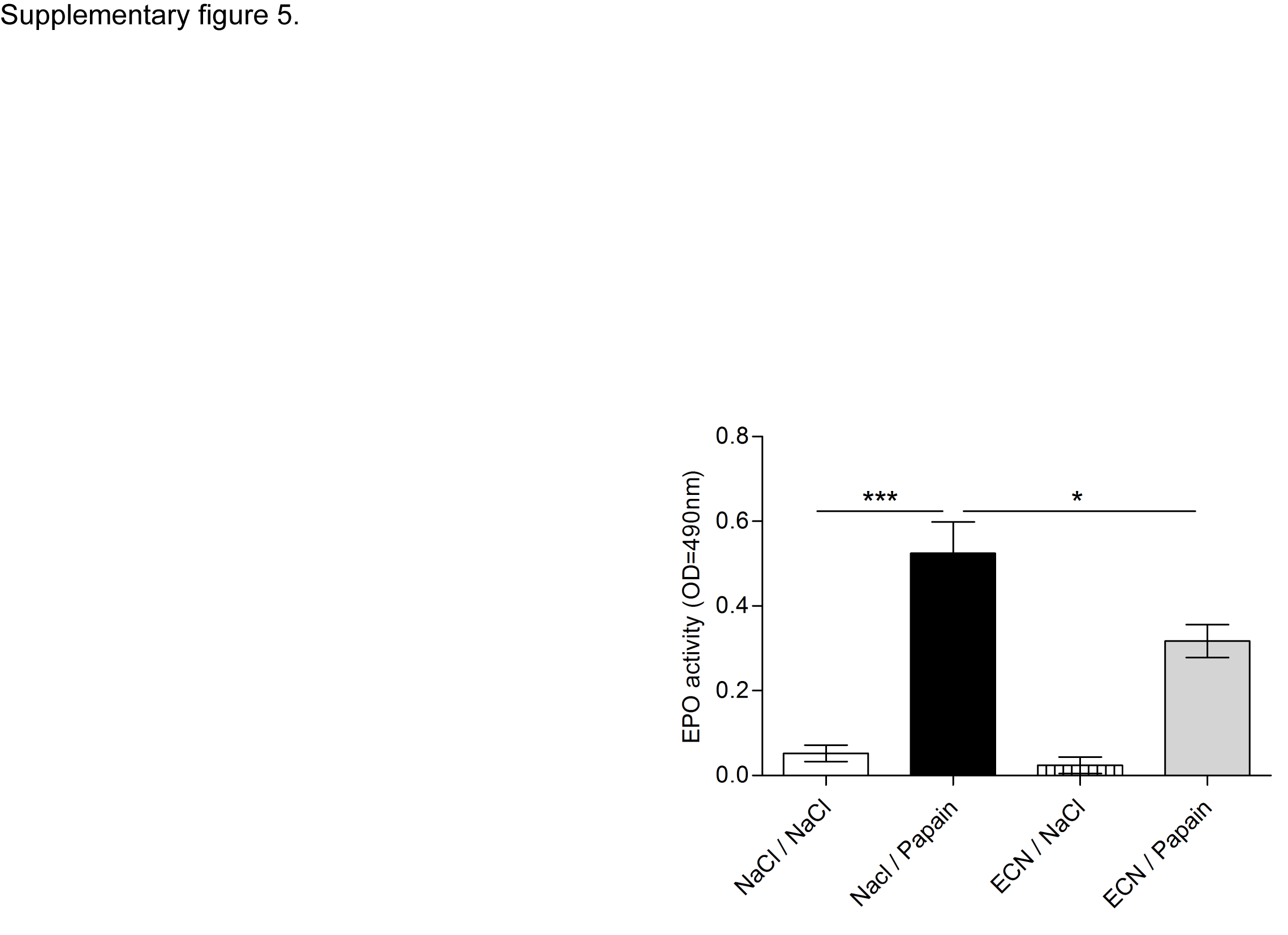


**
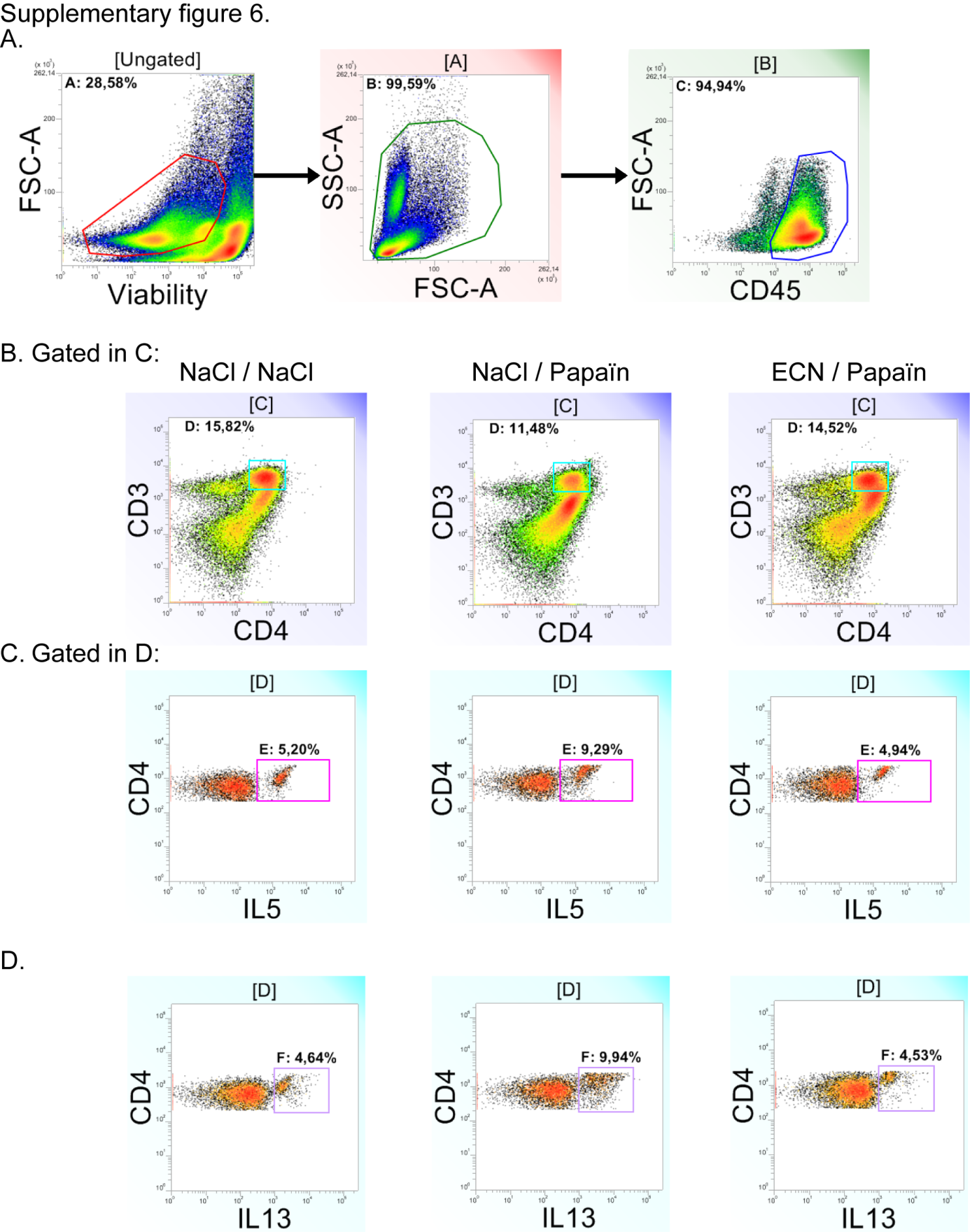
**

**
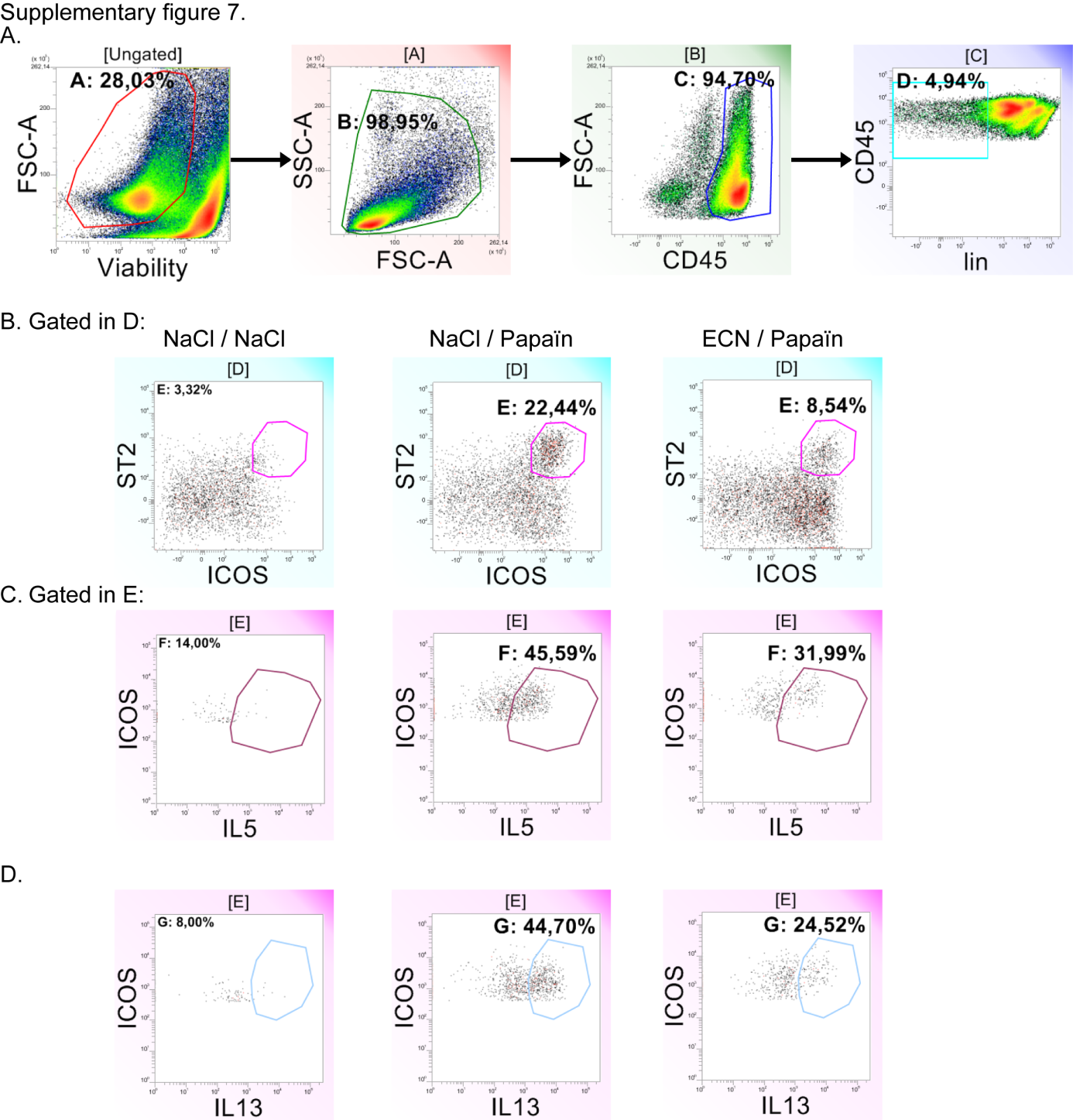
**

**Supplementary figure legends**

**Supplementary figure 1.**

MPO activity was determined in lung homogenates. Data are expressed as mean + SEM from a single experiment representative of 2 experiments with n=5 mice per group. The parametric one-way ANOVA test with multiple Bonferroni’s comparison test was used. *** refer to *P*<0.001.

**Supplementary figure 2.**

(A) Experimental settings of acute papaïn-induced lung inflammation and MG1655 treatment. (B) Total cells and differential cell count of eosinophils, neutrophils, lymphocytes and macrophages were determined in BALF by numeration of MGG stained cytospin. BALF levels of (C) CXCL1 and (D) MPO were measured by ELISA. Lung homogenate levels of (E) CCL11 and (F) CCL17 were measured by ELISA. Data are expressed as mean + SEM from a single experiment representative of 2 experiments with n=5 mice per group. The parametric one-way or two-way ANOVA test with multiple Bonferroni’s comparison test was used. *, ** and *** refer to *P*<0.05, *P*<0.01 and *P*<0.001, respectively.

**Supplementary figure 3.**

(A) Experimental settings of chronic papaïn-induced lung inflammation and MG1655 treatment. (B) Lung tissues were histologically examined 24 h after the last papaïn challenge. Lung sections strained with HE from controls (NaCl/NaCl), papaïn (NaCl/Papaïn) and MG1655 (MG1655/Papaïn)-treated mice are represented. (C) Histological score of lung inflammation infiltration was performed on paraffin embedded section after HE staining. (D) Histological score of airway remodeling was performed on paraffin embedded section after HE staining. (E) Histological score of lung mucus production was performed on paraffin embedded section after PAS staining. (F) Total cells and differential cell count of eosinophils, neutrophils, lymphocytes and macrophages were determined in BALF by numeration of MGG stained cytospin. Lung homogenate level of (G) CCL11, (H) CCL17 and (I) CXCL1 were measured by ELISA. Data are expressed as mean + SEM from a single experiment representative of 2 experiments with n=5 mice per group. The parametric one-way or two-way ANOVA test with multiple Bonferroni’s comparison test was used. *, ** and *** refer to *P*<0.05, *P*<0.01 and *P*<0.001, respectively.

**Supplementary figure 4.**

Evaluation of gut colonization in feces homogenate during chronic papain-lung inflammation in animals treated with ECN (A and B) and MG1655 (C and D). Enterobacteria were isolated and evaluated on McConkey-agar medium (A and C) and *E. coli* were isolated and evaluated on McConkey-agar supplemented with 50µg/mL of streptomycin (B and D). Data are expressed as mean + SEM from a single experiment representative of 2 experiments with n=5 mice per group.

**Supplementary figure 5.**

EPO activity was determined in lung homogenates. Data are expressed as mean + SEM from a single experiment representative of 2 experiments with n=5 mice per group. The parametric one-way ANOVA test with multiple Bonferroni’s comparison test was used. * and *** refer to *P*<0.05 and *P*<0.001, respectively.

**Supplementary figure 6.**

Gating strategy used to identify CD4+ T lymphocytes in mouse lung. (A) Cells were isolated from enzymatically digested mouse lungs, and after the exclusion of dead cells, immune cells were identified by CD45 staining. (B) A sequential gating strategy was first used to identify populations expressing T lymphocyte markers CD3 and CD4. (C) Identification of IL5 and (D) IL13 producing CD3+ CD4+ (expressed as % of parental gate).

**Supplementary figure 7.**

Gating strategy used to identify ILC2 cells in mouse lung. (A) Cells were isolated from enzymatically digested mouse lungs, and after the exclusion of dead cells, immune cells were identified by CD45 staining. (B) A sequential gating strategy was first used to identify populations not expressing lineage markers (B220, FcεRI, CD11b, CD3ε and Siglec F) while expressing ICOS and ST2. (C) Identification of IL5 and (D) IL13 producing ILC2 (expressed as % of parental gate).
